# Automated Blood Cell Detection and Classification in Microscopic Images Using YOLOv11 and Optimized Weights

**DOI:** 10.3390/diagnostics15010022

**Published:** 2024-12-25

**Authors:** Halenur Sazak, Muhammed Kotan

**Affiliations:** Department of Information Systems Engineering, Faculty of Computer and Information Sciences, Sakarya University, Sakarya 54050, Turkey; mkotan@sakarya.edu.tr

**Keywords:** YOLOv11, blood cell detection, automated detection, medical imaging, computer vision

## Abstract

**Background/Objectives:** Accurate detection and classification of blood cell types in microscopic images are crucial for diagnosing various hematological conditions. This study aims to develop and evaluate advanced architectures for automating blood cell detection and classification using the newly proposed YOLOv10 and YOLOv11 models, with a specific focus on identifying red blood cells (RBCs), white blood cells (WBCs), and platelets in microscopic images as a preliminary step of the complete blood count (CBC). **Methods**: The Blood Cell Count Detection (BCCD) dataset was enriched using data augmentation techniques to improve model robustness and diversity. Extensive experiments were performed, including complete weight initialization, advanced optimization strategies, and meticulous hyperparameter tuning for the YOLOv11 architecture. **Results**: The YOLOv11-l model achieved an overall mean Average Precision (mAP) of 93.8%, reflecting its robust accuracy across multiple blood cell types. **Conclusions**: The findings underscore the efficacy of the YOLOv11 architecture in automating blood cell classification with high precision, demonstrating its potential to enhance hematological analyses and support clinical diagnosis.

## 1. Introduction

Identifying blood cell types is essential for diagnosing and managing various hematological diseases, including leukemia and lymphoma. Microscopic examination of blood cells remains crucial in detecting morphological abnormalities and unusual cell count increases, which are vital for early intervention and disease management [1]. Such analyses are indispensable for pinpointing cancer subtypes, determining the severity of hematological disorders, and crafting personalized treatment strategies. Automating blood cell analysis can significantly expedite and improve diagnostic processes, driving innovations in medical technology.

Blood cells can be categorized into three main types: erythrocytes (red blood cells), leukocytes (white blood cells), and platelets. Erythrocytes are responsible for transporting oxygen from the lungs to the body and returning carbon dioxide to the lungs. Their biconcave shape increases surface area for gas exchange, ensuring sufficient oxygen levels are maintained in the blood. Leukocytes defend the body against infections and diseases as part of the immune system. These cells include various types, such as neutrophils, lymphocytes, monocytes, eosinophils, and basophils. Abnormalities in leukocyte counts, particularly elevated levels in leukemia, are critical indicators of underlying health issues. On the other hand, platelets are tiny cell fragments essential for blood clotting and the healing of wounds. They help prevent bleeding by clumping together at the site of injury; however, low platelet counts (thrombocytopenia) can lead to excessive bleeding, while high counts can increase the risk of thrombosis [1].

In diseases such as anemia and thrombocytopenia, the quantitative analysis of erythrocytes and platelets is essential for diagnosis. A decrease in erythrocyte count in anemia indicates a reduction in the body’s oxygen-carrying capacity, while thrombocytopenia, characterized by a platelet deficiency, can lead to clotting disorders. Accurate and timely diagnosis of such conditions is vital for improving treatment outcomes and better managing the course of the disease [2].

The manual detection and classification of blood cells is time-consuming and tends to produce subjective results, as it relies on the observer’s experience and attention. Additionally, this method is impractical for large datasets and commonly leads to inconsistencies among healthcare professionals [3].

In recent years, advancements in computer vision and deep learning techniques have created significant opportunities for the automation of medical imaging. Convolutional Neural Networks (CNNs) and other artificial intelligence (AI) techniques have great potential in predicting and classifying blood cells, enabling more accurate and faster diagnoses by analyzing complex patterns in medical images. Integrating AI-based approaches and deep learning models into blood cell classification studies aligns with current research trends and contributes to a deeper understanding of machine learning applications in healthcare [4,5].

AI substantially impacts blood cell detection, enhancing the precision and speed of diagnostics. AI algorithms can learn from vast quantities of data, identifying patterns and anomalies that human observers may overlook. Deep learning-based object detection methods have undergone rapid evolution, offering more efficient and accurate solutions compared to traditional approaches, and continue to do so. Among these methods, the You Only Look Once (YOLO) model stands out due to its ability to balance speed and accuracy. While earlier object detection algorithms, such as R-CNN, required a multi-stage processing model, YOLO can detect multiple objects in a single step, making it particularly advantageous for real-time applications.

Although partially automated systems for blood cell analysis are employed in some clinical settings, their accuracy and processing speed often need improvement. YOLO-based approaches have the potential to overcome these limitations with their superior speed and accuracy, offering more reliable and efficient solutions for clinical diagnostics. Using AI in blood cell detection, healthcare professionals can achieve faster turnaround times for results, facilitating timely interventions and improved patient outcomes.

The primary research problem in this study is the need for more efficient and scalable blood cell detection and classification methods. Numerous studies have demonstrated the success of deep learning-based object detection algorithms in microscopic blood cell imagery [6]. Although current AI-based approaches show promise, achieving high accuracy and real-time performance in microscopic blood cell imaging remains a critical challenge. To address this, this study evaluates the latest YOLO architectures, specifically YOLOv10 and YOLOv11, which offer enhanced architectures and optimization techniques leading to high accuracy rates and fast inference. These models have great potential for applications in medical imaging, particularly in detecting microscopic blood cells.

In this study, we evaluate the performance of YOLOv10 and YOLOv11 models and their weights on the Blood Cell Count Detection (BCCD) dataset [7], which consists of a diverse collection of microscopic blood cell images. The morphological variability of the BCCD dataset provides an ideal testing ground for classification challenges. Through experiments on microscopic blood cell images, we examine how these models contribute to medical image processing, especially in the context of blood cell detection. The main contributions of this research are as follows: (1) investigating the performance of YOLOv10 and YOLOv11 on the BCCD dataset, which presents significant morphological diversity and classification challenges, (2) conducting a comparative analysis of these models to evaluate their strengths and weaknesses in microscopic blood cell detection, and (3) demonstrating the applicability of recent YOLO-based object detection methods in clinical diagnostics to address the critical need for speed and accuracy in blood cell analysis. These findings offer valuable insights into the potential integration of deep learning techniques in healthcare, paving the way for improved patient outcomes through AI-driven diagnostics.

The remainder of this paper is organized as follows: Section 2 reviews related work in blood cell detection and classification using deep learning techniques. Section 3 describes the materials and methods used in this study, including data augmentation strategies and the details of implementing the YOLO models. In Section 4, we present the model’s experimental results and performance evaluations. Section 5 discusses the findings, their implications for clinical practice, and potential limitations of the study. Finally, Section 6 concludes the paper by summarizing the key contributions.

## 2. Literature Review

Blood cell detection using deep learning has emerged as a pivotal area of research within medical imaging, primarily due to the ability of deep learning models to automate and enhance the accuracy of cell classification and diagnosis. Various studies including CNNs, Recurrent Neural Networks (RNNs), and transfer learning have demonstrated the effectiveness of deep learning techniques in identifying different types of blood cells, including white blood cells (WBCs) and red blood cells (RBCs), as well as detecting abnormalities such as cancer and malaria.

For instance, Patil et al. [8] utilized publicly available databases for blood cell detection, proposing that a hybrid CNN and RNN model achieved a high success rate through a canonical correlation analysis. Hegde et al. [9] compared traditional image processing approaches with deep learning methods for classifying white blood cells. The application of CNNs in blood cell detection is well established, having been employed to classify WBCs and to detect conditions such as sickle cell anemia and leukemia. Research indicates that CNNs can effectively capture intricate spatial patterns in blood cell images, which is crucial for distinguishing between various cell types and detecting abnormalities [10]. The architecture of CNNs allows for automatic feature extraction, significantly reducing the need for manual intervention and enhancing diagnostic efficiency [11]. Furthermore, hybrid models that combine CNNs with other deep learning techniques have shown promise in analyzing blood cell images over time, thereby improving classification performance [12].

In addition to CNNs, other deep learning frameworks have been explored for blood cell detection. For example, ensemble methods have been proposed to mitigate the overfitting issues commonly associated with CNNs, particularly in detecting malaria parasites in blood smear images [13]. Moreover, advanced techniques such as squeeze-and-excitation networks have enhanced feature representation in detecting leukemia from microscopic blood samples [14]. These innovations highlight the versatility and adaptability of deep learning models in addressing the challenges posed by the complex nature of blood cell images.

The availability of large datasets has also played a crucial role in advancing deep learning applications in blood cell detection. Datasets such as the Raabin-WBC dataset provide a wealth of annotated images that facilitate the training and validation of deep learning models, thereby improving their generalization capabilities [15]. Integrating these datasets with deep learning algorithms has significantly improved the accuracy and speed of blood cell classification, which is essential for timely diagnosis and treatment [16]. The continuous advancements in deep learning architectures, coupled with the availability of extensive datasets, are likely to further enhance these models’ capabilities in clinical settings, ultimately improving patient outcomes through more accurate and efficient diagnostic processes.

The application of the YOLO framework for blood cell detection has gained significant traction in recent years due to its efficiency and accuracy in identifying various blood components. YOLO’s architecture allows for real-time object detection, which is particularly beneficial in clinical settings where timely diagnosis is critical. Studies have demonstrated that YOLO can achieve high accuracy rates in detecting RBCs, WBCs, and platelets (PLTs) [17].

Recent advancements in YOLO variants, such as YOLOv4 and YOLOv8, have also contributed to improved performance in blood cell detection. Mustaqim et al. [18] utilized a combination of Cross Stage Partial Network and GhostNet with Spatial Pyramid Pooling on YOLOv4 to detect acute lymphoblastic leukemia subtypes in multi-cell blood images, showcasing the adaptability of YOLO to complex medical imaging tasks. Similarly, Nugraha [19] integrated YOLOv8 with Detection Transformer (DETR), further improving the accuracy of white blood cell detection, emphasizing the continuous evolution of YOLO-based methodologies. These developments highlight the importance of integrating advanced neural network architectures to enhance the performance of YOLO in medical applications.

Moreover, improved YOLO variants have addressed the challenges associated with overlapping blood cells and bounding-box positioning. Automating this process using YOLO-based systems can significantly reduce the workload on healthcare professionals and minimize human error in blood analysis [20]. Jiang et al. [12] proposed an attention-guided deep learning method that enhanced detection performance by focusing on the spatial relationships between overlapping cells, a common issue in blood cell imaging. That approach not only improved the accuracy of detection but also aided in the counting of blood cells, which is crucial for diagnosing various hematological conditions. Yücel and Çetintaş [21] aimed to develop an automatic system for blood cell classification using the YOLOv9 architecture and the BCCD dataset [7]. Different optimization algorithms and learning rates were observed in the developed scenario with YOLOv9 architecture. A success rate of approximately 92% was achieved. Xu et al. [22] proposed a lightweight model based on Tiny and Efficient YOLOF (TE-YOLOF) to solve the problem of the low detection sensitivity of red blood cells and to increase the overall detection accuracy. It was shown that fewer parameters provided a better success rate with this model. Liu et al. [23] proposed the idea of an attention mechanism and developing a squeeze-and-excitation-based YOLO-v3 detection model (ISE-YOLO). In that model, the improved SE module was added to different structural blocks of YOLO. Experimental results showed that the proposed ISE-YOLO model improved by 96.5% on WBCs, 92.7% on RBCs, and 89.6% on platelets. Wang et al. [24] proposed YOLO-FMS, a lightweight and efficient model based on YOLOv5. According to the experimental results, YOLO-FMS had a mean Average Precision (mAP) of 92.5% on the BCCD dataset and 87.6% on the Tuberculosis-Phonecamera dataset. In another study, Mao et al. [25] developed the DWS-YOLO model for blood cell detection. That model included several innovative modules, such as a lightweight C3 module, improved joint attention mechanism, the Scylla-IoU loss function, and improved soft non-maximum suppression. The improved attention mechanism, loss function, and suppression techniques increased the detection accuracy, while the lightweight C3 module reduced the computational time.

The continuous development of YOLO architectures and the integration of advanced techniques such as attention mechanisms and transformative models can potentially increase the accuracy and reliability of blood cell analysis in clinical settings. The literature review shows that many studies have been conducted with classical algorithms, but studies on YOLO are more limited. The most recent of these studies are those using the YOLOv9 algorithm. In this study, blood cell detection was performed using all the weights of the YOLOv10 and YOLOv11 algorithms, and a comparative analysis was performed with high success rates. Table 1 summarizes and compares the performance of recent studies on YOLO-based blood cell detection using the BCCD dataset. It highlights the advancements achieved by different models, including the recently developed YOLOv10-l and YOLOv11-l models. The YOLOv11-l model especially stands out with an accuracy of 91.8% for the “Platelets” class, along with high accuracy rates of 90.2% in the detection of RBCs and 99.0% in the detection of WBCs.

## 3. Materials and Methods

This study performed a comprehensive and comparative analysis using YOLOv10 and YOLOv11 architectures for blood cell detection. The BCCD dataset, which formed the basis of the research, provides images containing RBCs, WBCs, and platelets. Within the scope of the study, BCCD data consisting of augmented images were used to increase the training success in the dataset. The dataset, applied methods, and design details are presented in the following.

### 3.1. Dataset

**Figure 1:**
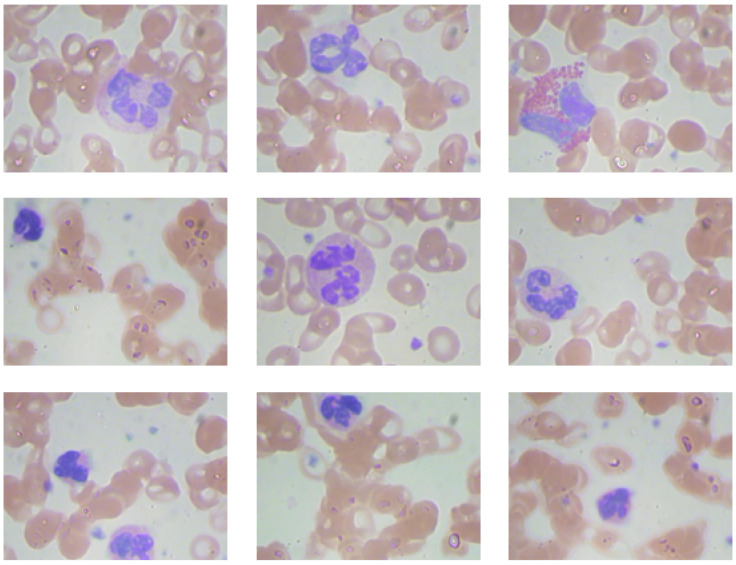
Sample images from the BCCD dataset.

The BCCD dataset [7] is a microscopic dataset used in this study. Figure 1 presents sample images from the dataset. It contains images of various types of blood cells, including platelets (thrombocytes), leukocytes (white blood cells), and erythrocytes (red blood cells). There are 364 images across three classes: WBC, RBC, and platelets. A total of 4888 labels are assigned to these classes. This dataset is frequently used to train and test deep learning algorithms for diagnosing hematological diseases, focusing on detecting and classifying blood cells in microscopic images.

The BCCD dataset is particularly valuable for blood cell detection and classification research, as it offers a rich collection of images gathered from various laboratory settings. This dataset serves as a fundamental resource for research on the automated analysis of blood cells, providing an effective platform to evaluate the performance of deep learning algorithms in object detection and classification tasks.

#### Data Augmentation

Various augmentation techniques were applied to the BCCD dataset to enhance the overall performance of the model and prevent overfitting. The flip operation was used to transform the images horizontally and vertically, strengthening the model’s ability to recognize symmetric objects. The 90° rotation operation allowed the model to detect objects from different angles by rotating images clockwise, counterclockwise, and upside down. The crop operation facilitated cutting images at various zoom levels, contributing to the model’s ability to learn different-sized objects more effectively. The hue, saturation, brightness, and exposure augmentations applied for color and lighting adjustments changed the color tones, saturation levels, and brightness of the images, thereby increasing the model’s resilience to varying lighting conditions. These techniques enhanced the overall diversity of the model, leading to a more robust and generalizable learning process [27]. As a result of applying the augmentation techniques, the dataset had 875 images for three classes.

### 3.2. YOLOv10

YOLO has emerged as a leading architecture in real-time object detection by establishing an effective balance between high performance and efficiency. Continuous innovations are being made in architectural designs and optimization objectives for YOLO models. However, the dependency on Non-Maximum Suppression (NMS) during the post-processing phase limits the end-to-end usability of these models and creates latency issues, negatively impacting overall performance. This next-generation model addresses the shortcomings found in previous YOLO versions, achieving an effective balance between performance and efficiency. By eliminating dependence on NMS and optimizing various model components, YOLOv10 achieves high performance with significantly reduced computational load.

In YOLOv10, significant improvements were made in model architecture and post-processing steps to overcome these limitations. Significantly, a new consistent dual assignment method was developed, eliminating the need for NMS. That approach enabled high accuracy while reducing inference latency, ensuring efficient end-to-end model operation.

YOLOv10 stands out as a model that offers similar or higher performance than its previous versions, with fewer parameters and reduced latency. Extensive evaluations show superior accuracy–latency balance among multiple model variants, including YOLOv10-n, YOLOv10-s, YOLOv10-m, YOLOv10-b, YOLOv10-l, and YOLOv10-x. For instance, YOLOv10-s operates 1.8 times faster than the RT-DETR-R18 model on the COCO dataset while maintaining a similar average precision (AP) value [28]. On the other hand, YOLOv10-b exhibits 46% lower latency and 25% fewer parameters compared to YOLOv9-c at the same performance level.

**Figure 2:**
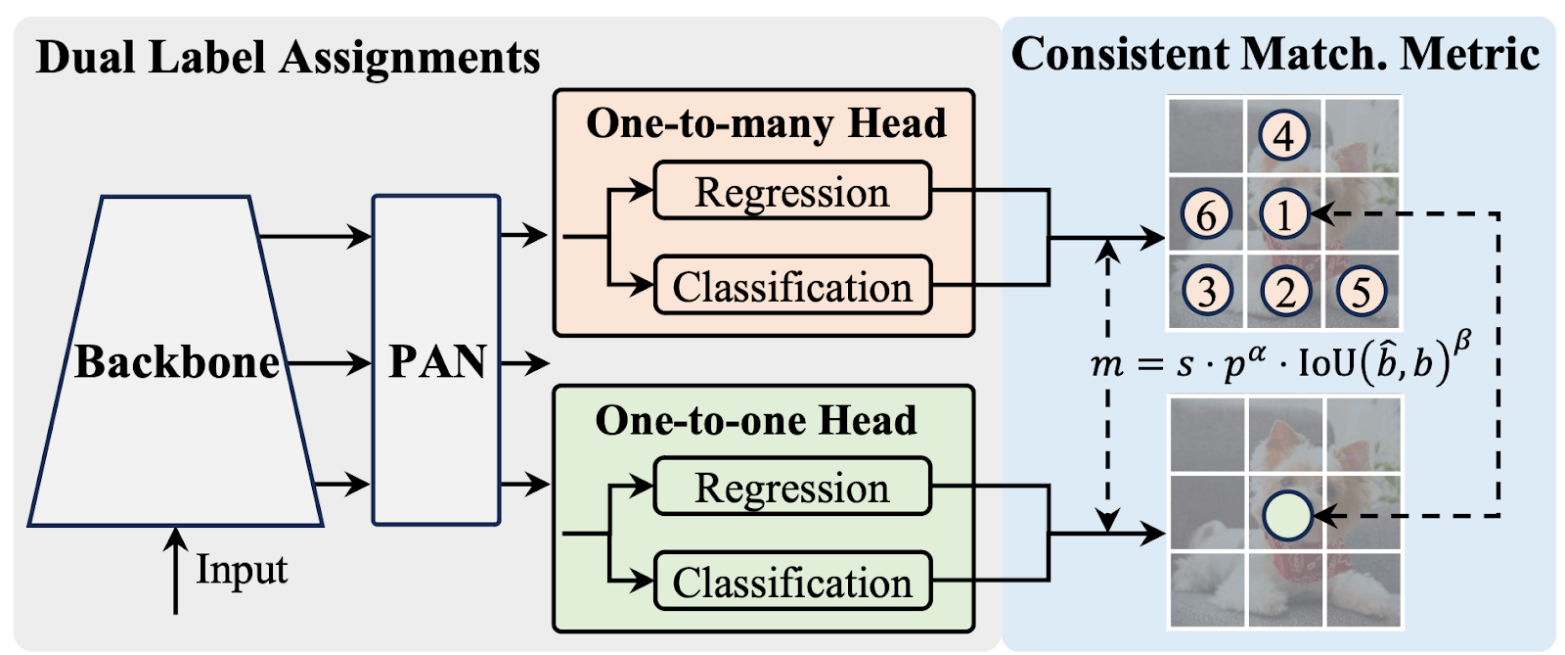
Consistent dual assignments for NMS-free training [29].

The architecture of YOLOv10 features a Cross Stage Partial Network (CSPNet)-based backbone designed with Path Aggregation Network (PAN) layers to collect features at different scales, multiple heads to provide rich supervision signals during training, and a one-to-one head designed to eliminate the need for NMS during inference. This architecture is shown in Figure 2. These components are integrated within a holistic model design strategy emphasizing efficiency and accuracy. These innovations enable YOLOv10 to deliver impressive performance while maintaining high efficiency, establishing it as a contemporary solution in object detection.

### 3.3. YOLOv11

YOLOv11 is the latest version of real-time object detection. This latest release offers significant advancements in speed and efficiency. It adds substantial improvements over previous versions. An advanced backbone and neck architecture provides more precise and effective feature extraction capabilities. This increases the capacity to handle complex tasks, allowing object detection to be performed more accurately.

One of the key advancements in YOLOv11 is the introduction of the C2PSA (Cross-Stage Partial with Self-Attention) module in the backbone. This module integrates the strengths of cross-stage partial networks with self-attention mechanisms, allowing the model to capture contextual information across multiple layers more effectively. As a result, YOLOv11 demonstrates enhanced accuracy, particularly when detecting small or occluded objects. Another significant improvement is replacing the C2f block with C3k2 in the backbone and head, a custom CSP Bottleneck implementation. Unlike YOLOv8’s single large convolution, C3k2 employs two smaller convolutions, striking a balance between accuracy, efficiency, and processing speed [30].

Enhanced training pipelines and well-tuned architectural designs contribute to YOLOv11’s superior processing speed, achieving an excellent balance between performance and precision. Notably, the YOLOv11-m model achieved a higher average mAP (mean Average Precision) while using 22% fewer parameters on the COCO dataset [31]. This ensures that the model remains computationally efficient while maintaining accuracy levels.

Additionally, YOLOv11’s versatility allows seamless deployment across various environments, including edge devices, cloud platforms, and systems equipped with NVIDIA GPUs. This flexibility makes it suitable for a broad spectrum of applications, ranging from object detection and instance segmentation to image classification, pose estimation, and oriented bounding box (OBB) detection.

Overall, YOLOv11 is a powerful option for computer vision tasks in academic and industrial domains. Its innovative architecture addresses diverse challenges in the field, offering adaptable and high-performing solutions for real-time detection and related applications.

### 3.4. Evaluation Metrics

The core performance metrics used to evaluate machine learning models are classification accuracy and more sensitive indicators that measure model success [32]. When comparing accurately predicted positive (*TP*) and negative (*TN*) samples to all samples (*TP* + *TN* + *FP* + *FN*), accuracy is measured. *FP* (False Positive) refers to cases where the model incorrectly classifies a non-existent object as positive. In contrast, *FN* (False Negative) refers to instances where the model fails to detect an existing object. *TP* (True Positive) indicates cases where the model correctly identifies an existing object, and *TN* (True Negative) denotes when the model correctly rejects a non-existent object. Equation (1) expresses this ratio as follows:

(1) $$\mathrm{Accuracy}=\frac{TP+TN}{TP+TN+FP+FN}$$

However, accuracy is not always a reliable metric, particularly when dealing with unbalanced datasets. As a result, additional metrics offer a more thorough assessment of the model’s performance. Precision is a metric that indicates the proportion of predicted positive samples that are correctly classified. Equation (2) defines precision as:

(2) $$\mathrm{Precision}=\frac{TP}{TP+FP}$$

Another critical metric, recall, shows how well the model correctly identifies true positive samples. Recall is calculated using Equation (3):

(3) $$\mathrm{Recall}=\frac{TP}{TP+FN}$$

The *F*1 score, which measures the balance between precision and recall, is the harmonic mean of these two metrics and is particularly important in imbalanced datasets. The *F*1 score is defined by Equation (4):

(4) $$F1=2\times \frac{\mathrm{Precision}\times \mathrm{Recall}}{\mathrm{Precision}+\mathrm{Recall}}$$

Model performance is evaluated for more complex problems like object detection using average precision (AP), calculated as the area under the precision–recall curve (AUC). AP is expressed by Equation (5):

(5) $$\mathrm{AP}=\int_{0}^{1}P\left(R\right)\;dR$$

To evaluate model performance at a specific IoU (Intersection over Union) threshold, mAP@50 is used, which averages the AP values calculated at an IoU threshold of 0.50. This metric is a commonly used method for evaluating the accuracy of the model and is defined by Equation (6). AP50 is the average precision value calculated at the 0.50 IoU (Intersection over Union) threshold for each class, and *N* represents the number of classes.

(6) $$\mathrm{mAP}@50=\frac{1}{N}\sum_{i=1}^{N}AP_{50,i}$$

To assess model performance at different IoU (Intersection over Union) thresholds, the mAP@50-95 metric is used. This metric takes the average of AP values calculated at IoU thresholds ranging from 0.50 to 0.95 (in 0.05 increments). mAP@50-95 is represented by Equation (7):

(7) $$\mathrm{mAP}@50-95=\frac{1}{10}\sum_{k=1}^{10}\left(\frac{1}{N}\sum_{i=1}^{N}AP_{k,i}\right)$$

While accuracy, precision, recall, and *F*1 score are critical metrics for evaluating machine learning model performance, advanced metrics like mAP provide a more comprehensive assessment, especially for object detection models [33,34].

## 4. Experimental Results

In this study, after performing data augmentation on the BCCD dataset, object detection was carried out using YOLOv10 and YOLOv11 models. The models were trained on a Tesla T4 GPU with an NVIDIA driver version 535.104.05 and CUDA 12.2. The models were trained using the weights n,s,x,l,m,b for YOLOv10, while only n,s,x,l,m were utilized for YOLOv11. These weights correspond to the following: *n* (nano), *s* (small), *x* (extra-large), *l* (large), *m* (medium), and *b* (balanced). The different weights (*m, n, x, s, l, b*) used in the YOLOv10 and YOLOv11 models are selected to balance computational efficiency and model accuracy. These variants demonstrate the model’s adaptability by offering solutions tailored to diverse application needs [29].

A hold-out validation approach was employed for model validation, with the dataset split into training and test sets at an 80/20 ratio. The training set was utilized for model optimization and hyperparameter tuning, while the test set was reserved exclusively for evaluating the final performance of the trained model. This approach ensured an accurate assessment of the model’s generalizability to unseen data.

During model optimization, the most suitable optimization settings were automatically determined. The AdamW optimizer was utilized with a learning rate of lr = 0.001429 and a momentum value of momentum = 0.9. AdamW was selected due to its proven advantages during the training process, including its effectiveness on sparse gradients and strong regularization capabilities, as supported by prior studies [35,36,37].

**Figure 3:**
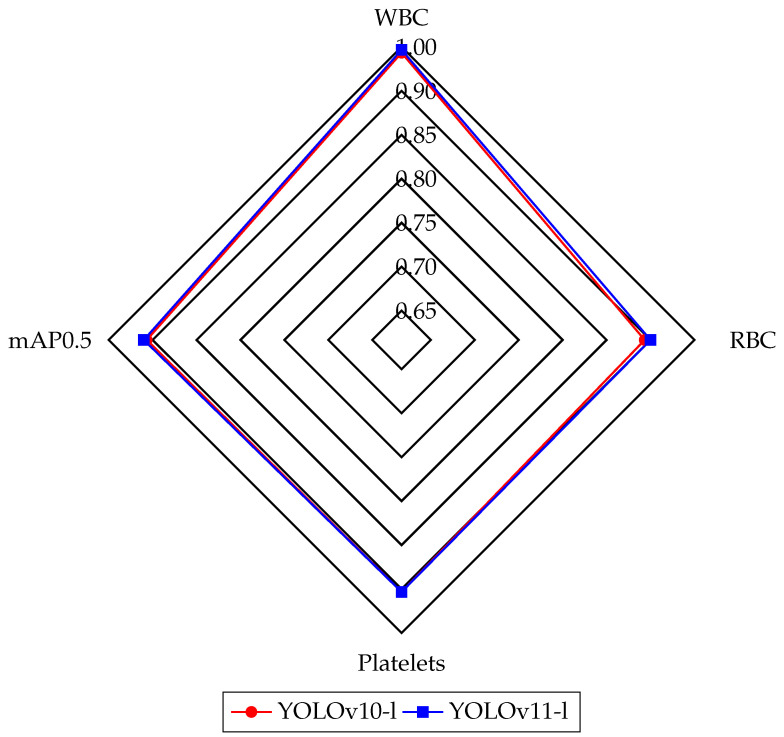
Radar chart comparing performance metrics (WBC, RBC, Platelets, mAP@0.5) for YOLO models: YOLOv10-l and YOLOv11-l.

The results of the training runs are presented in Table 2 and Table 3, where the performance of the YOLOv10 and YOLOv11 models is highlighted. YOLOv10-l outperformed other versions on the mAP50 (0.927), while YOLOv10-m achieved high precision at 0.874. In the case of YOLOv11, YOLOv11-l achieved one of the highest mAP50 (0.938) and performed well on the mAP50-95 (0.663), compared to models like DWS-YOLO (mAP50: 0.938) and YOLO-FMS (mAP50: 0.925). Furthermore, YOLOv11-l excelled in precision (0.873), demonstrating its ability to provide more accurate positive predictions by minimizing false positives. Overall, YOLOv10 and YOLOv11 models performed on par with, or ahead of, top-performing models from the literature, confirming their strong overall performance. YOLOv11 performed better than YOLOv10 regarding mAP. The advanced architecture of YOLOv11 contributed to higher detection accuracy, with a particularly noticeable improvement in the detection and classification of blood cells. In both YOLOv10 and YOLOv11 models, the l weight yielded the best results with respect to mAP50. We compared the best-performing weights of the YOLOv10-l and YOLOv11-l models using a radar chart illustrating their performance metrics on the BCCD dataset classes—WBC, RBC, platelets—and the mAP@0.5, as shown in Figure 3. This comparison highlights the effectiveness of the newer YOLO version in medical imaging analysis tasks, especially in detecting microscopic blood cells.

**Figure 4:**
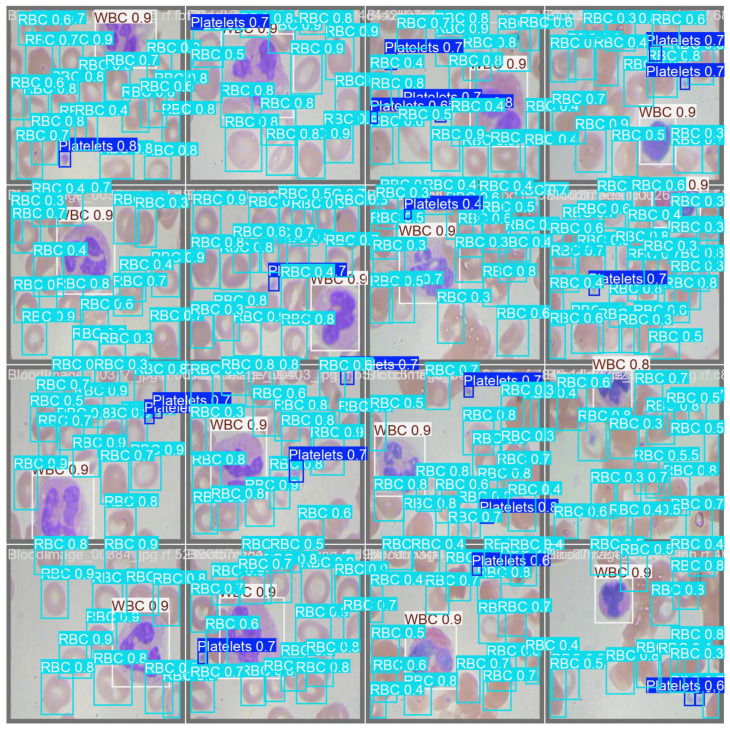
Demonstration of blood cell detection with YOLOv11-l.

**Figure 5:**
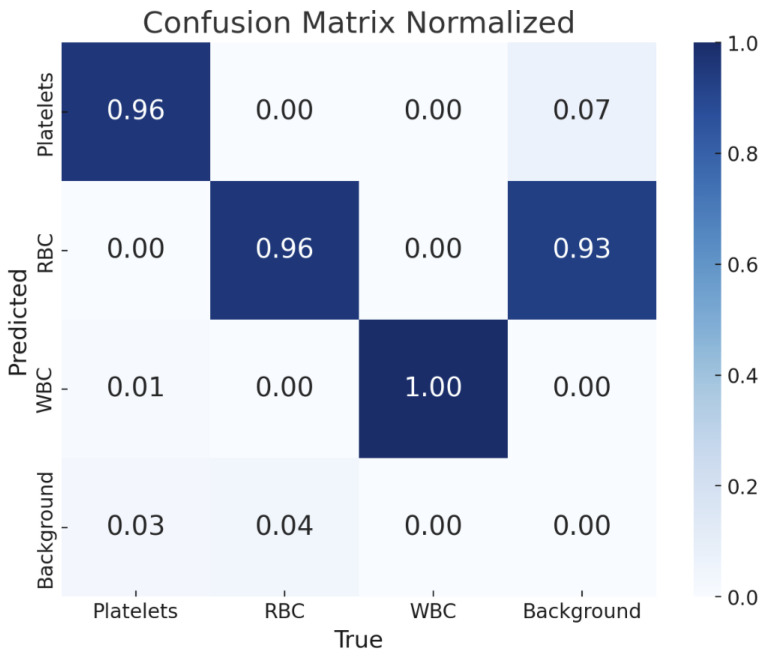
Confusion matrix for YOLOv11-l.

Figure 4 shows the detection results of the YOLOv11-l weight. The confusion matrix is also presented in Figure 5, providing a detailed overview of the model’s classification performance across different blood cell types and the background class. Each cell within the matrix indicates the proportion of predictions that fall into each category, normalized to account for the distribution of classes. The matrix reveals that the YOLOv11-l model achieved high accuracy in classifying WBCs, with a perfect score of 1.00, demonstrating its exceptional capability in correctly identifying that cell type. Platelets and RBCs were also classified with high precision, both scoring 0.96.

**Figure 6:**
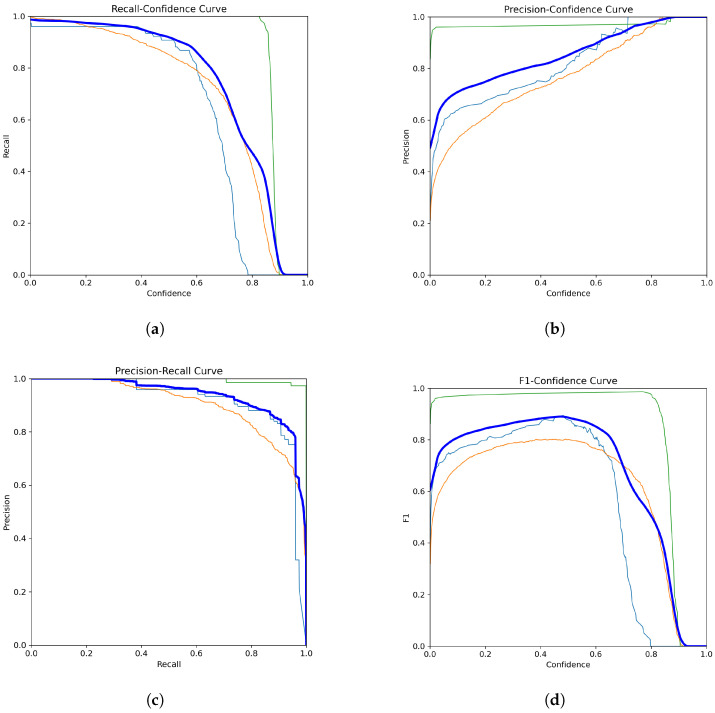
Performance curves for YOLOv11-l in blood cell detection and classification. (**a**) Recall vs. Confidence, (**b**) Precision vs. Confidence, (**c**) Precision vs. Recall, (**d**) F1 Score vs. Confidence.

**Figure 7:**
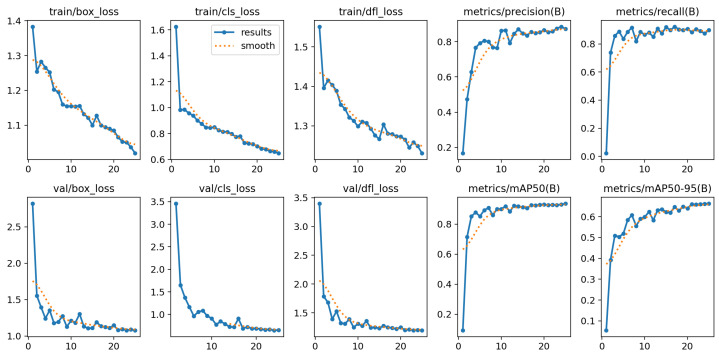
Results of YOLOv11-l model

Figure 6 and Figure 7 collectively demonstrate the YOLOv11-l model’s compelling performance in classifying blood cell types and its training progression over multiple epochs. Figure 6 illustrates the model’s strong classification capabilities, with key findings including a robust and stable recall and high precision. The green line corresponds to WBCs, the orange line represents RBCs, and the blue line indicates platelets. The thick dark blue line represents the average performance across all classes. Overall, the model performed reliably, especially in detecting WBCs. Figure 7 presents the training and validation metrics for the YOLOv11-l model over multiple epochs. Both training and validation losses decreased consistently, reflecting effective learning and generalization. Precision, recall, and mAP metrics improved rapidly during the initial epochs and stabilized at high values, indicating robust detection and classification of blood cells. These results affirmed the model’s reliability and effectiveness in automating blood cell detection and classification, highlighting its potential for clinical applications.

Our study built upon these advancements by employing the latest YOLOv11 architecture with optimized weights. As presented in Table 1, our YOLOv11-l model achieved an mAP of 93.8%, matching one of the highest reported performance. It demonstrated exceptional accuracy in detecting WBCs at 99.0% and platelets at 91.8%. It offered improved detection rates and maintained high accuracy across all cell types compared to previous models.

This study also explored how these models could contribute to clinical diagnostic processes by providing faster and more accurate results than traditional methods. Furthermore, our research aimed to underscore the potential of recent deep learning-based automatic detection models in biomedical fields, offering promising pathways for future developments in automated medical imaging. In addition, the insights gained from this study could be applied to other biomedical datasets, potentially improving diagnosis and treatment in a broader range of diseases.

## 5. Discussion

The findings presented in this study demonstrate the effectiveness of YOLOv10 and YOLOv11 architectures in automatic blood cell detection and classification. The 93.8% success rate of the YOLOv11-l model is a significant achievement in correctly identifying blood cells. This suggests that the advancements in YOLOv11, such as enhanced feature extraction and optimized anchor-free mechanisms, are particularly effective for medical imaging tasks that require high accuracy and reliability. The confusion matrix further illustrates that the model excels at minimizing misclassifications. However, some areas still allow refinement, particularly in distinguishing between closely related cell types like RBCs and platelets. The performance metrics, including mAP and F1 scores, demonstrate that YOLOv11-l can effectively handle variations in cell morphology, which is critical for robust clinical applications. The reduction in training and validation losses highlights the efficiency of the training process, facilitated by advanced optimization techniques and rigorous hyperparameter tuning. However, some limitations remain. Despite the model’s high overall accuracy, specific misclassification rates suggest that additional data augmentation strategies or ensemble approaches could further enhance performance. Additionally, while beneficial, reliance on the BCCD dataset may limit the model’s generalizability to other blood cell datasets with different staining techniques or imaging variations. Our study contributes to automated hematological analysis, offering a reliable method for classifying blood cells that could aid in faster and more accurate diagnostics. Nevertheless, future research should consider expanding the dataset and incorporating more diverse cell types to ensure the model’s robustness across a broader range of clinical scenarios. Additionally, future research could focus on integrating transfer learning techniques in similar datasets to optimize model performance. Another approach could involve using hybrid models that combine YOLO with other state-of-the-art architectures, such as Transformers, to improve success rates. In addition, explainable artificial intelligence (XAI) methods could provide more transparency in the model’s decision-making processes, increasing clinicians’ trust in AI-based solutions for blood cell detection and classification.

## 6. Conclusions

This study investigated newly developed YOLOv10 and YOLOv11 architectures for blood cell detection and classification. As a result of experiments performed on the BCCD dataset, application of data augmentation techniques, and testing of all weights, it was observed that YOLOv11 exhibited a high success rate of 93.8%. YOLOv11 provided better results, especially in detecting white blood cells and platelets, and reached higher accuracy rates than YOLOv10. In addition, it was determined that YOLOv11 showed superior performance compared to other studies in the literature. In addition to being the first research conducted with YOLOv11, this study demonstrated the effectiveness of YOLO architectures in the blood cell classification process. The results show that a fast and accurate automatic system that can replace manual processes used in medical image analysis is possible. It can contribute to further model development in future studies by applying it to larger datasets and different cell types. In addition, improved algorithms can also contribute to this development.

## Figures and Tables

**Table 1:** Literature comparison on YOLO-based blood cell detection.

Reference	Model	mAP	RBC	WBC	Platelets
[24]	YOLOv5-s	89.2	84.8	97.5	85.5
[24]	YOLOv8-s	91.0	86.2	97.4	89.5
[24]	YOLOv9-s	92.2	87.5	97.1	89.2
[24]	YOLO-FMS	92.5	89.0	97.2	91.3
[23]	ISE-YOLO	85.7	92.7	96.5	89.6
[25]	DWS-YOLO	93.8	91.7	99.1	90.6
[22]	TE-YOLOF	91.9	87.3	98.7	89.8
[26]	FED	89.9	80.4	98.9	90.3
Ours	YOLOv10-l	92.7	88.2	98.4	91.6
Ours	YOLOv11-l	93.8	90.2	99.0	91.8

**Table 2:** YOLOv10 performance results.

Weight	Precision	Recall	mAP50	mAP50-95
YOLOv10-n	0.764	0.882	0.895	0.631
YOLOv10-s	0.862	0.875	0.921	0.654
YOLOv10-x	0.852	0.897	0.918	0.646
YOLOv10-b	0.847	0.878	0.918	0.636
YOLOv10-l	0.872	0.886	0.927	0.64
YOLOv10-m	0.874	0.845	0.915	0.642

**Table 3:** YOLOv11 performance results.

Weight	Precision	Recall	mAP50	mAP50-95
YOLOv11-n	0.866	0.893	0.93	0.654
YOLOv11-s	0.859	0.910	0.936	0.66
YOLOv11-x	0.865	0.901	0.93	0.664
YOLOv11-l	0.873	0.899	0.938	0.663
YOLOv11-m	0.862	0.921	0.933	0.668

## Data Availability

Data are available by the authors on request.

## Abbreviations

The following abbreviations are used in this manuscript:

AI	Artificial intelligence
AP	Average precision
BCCD	Blood Cell Count Detection
CNN	Convolutional Neural Network
CSPNet	Cross Stage Partial Network
DETR	Detection Transformer
IoU	Intersection over Union
MAP	Mean Average Precision
PAN	Path Aggregation Network
RBC	Red blood cells
RNN	Recurrent Neural Network
WBC	White blood cells
YOLO	You Only Look Once
XAI	Explainable artificial intelligence
